# Skull Metastasis From Uterine Leiomyosarcoma, a Rare Presentation for a Rare Tumor: A Case Report and Review of the Literature

**DOI:** 10.3389/fonc.2020.00869

**Published:** 2020-06-16

**Authors:** Alessandro Rizzo, Maria Concetta Nigro, Vania Ramponi, Carmine Gallo, Anna Myriam Perrone, Pierandrea De Iaco, Giovanni Frezza, Damiano Balestrini, Maika Di Benedetto, Jarno Morbiducci, Maria Abbondanza Pantaleo, Margherita Nannini

**Affiliations:** ^1^Department of Specialized, Experimental and Diagnostic Medicine, S.Orsola-Malpighi Hospital, University of Bologna, Bologna, Italy; ^2^Department of Neurosurgery, Bellaria Hospital, Azienda USL-IRCCS Institute of Neurological Sciences, Bologna, Italy; ^3^Anatomic Pathology, Ospedale Bellaria, Bologna, Italy; ^4^Gynecologic Oncology Unit, S.Orsola-Malpighi Hospital, Bologna, Italy; ^5^Department of Radiotherapy, Bellaria Hospital, Bologna, Italy; ^6^Radiotherapy Unit, Azienda Ospedaliero Universitaria, Ospedali Riuniti Ancona, Ancona, Italy; ^7^ASUR Marche, Zona Territoriale 9, Macerata Hospital, Macerata, Italy; ^8^“Giorgio Prodi” Cancer Research Center, University of Bologna, Bologna, Italy; ^9^Medical Oncology Unit, S.Orsola-Malpighi University Hospital, Bologna, Italy

**Keywords:** uterine leiomyosarcoma, uterine sarcoma, skull metastasis, uterine cancer, uterine sarcoma, intracranial recurrence

## Abstract

Uterine leiomyosarcoma (uLMS) is a rare and aggressive malignancy with poor clinical outcomes. Even when localized, uLMS is associated with high rates of local and distant recurrences that are usually fatal. Common sites of recurrence are lung, liver, pelvic lymph nodes, and vertebral and long bones, though atypical patterns of recurrence have been described. Among them, intracranial recurrence appears as a rare finding, almost exceptional in skull and dura. We describe the case of a solitary skull metastasis from uLMS in a 39-year-old woman, which represents the third reported case of skull recurrence in literature. After multidisciplinary discussion, the patient underwent surgery and received adjuvant radiotherapy. After 4 months, she is currently alive, without evidence of extracranial disease. This case highlights the importance of suspecting and recognizing atypical and extremely rare metastasis to this region. We encourage the need for large case series in order to provide further information about cranial recurrences of uLMS taking into account the paucity of data currently available in literature and the frequently unpredictable behavior of this rare and highly lethal disease.

## Background

Uterine leiomyosarcoma (uLMS) is a rare malignancy accounting for 1–3% of all uterine cancers and approximately for 65% of all uterine sarcomas (US) (1). Most of the patients affected by uLMS present with advanced (metastatic or inoperable) disease, with poor prognosis, and short life expectancy (2). Currently, complete surgical resection is the only potentially curative treatment option for uLMS, where it represents the treatment of choice if the tumor is deemed resectable (3). Nevertheless, relapse rates are high in patients undergoing potentially curative resection. The risk of recurrence is between 50 and 70% and 5-year overall survival (OS) is <50% in early stages, even after radical resection (4). Although distant and local recurrences are a major issue after surgery, the role of postoperative treatment in uLMS remains controversial with no strong data currently supporting its use (5, 6). Lung, liver, peritoneal cavity, pelvic lymph nodes, vertebral and long bones are typically reported sites of recurrent uLMS (7). The array of tools currently available in imaging techniques and the prompt detection in high-risk patients has allowed the identification of more atypical patterns of uLMS recurrence, such as heart, parotid gland, gastrointestinal tract, brain, and skull (8–10).

We herein report a case of cranial recurrence of uLMS in a young woman, representing to our knowledge the third case of uLMS skull metastasis reported in literature.

## Case Presentation

In January 2018, a 39-year-old woman presented to her gynecologist with menorrhagia and pelvic pain; her past medical history included uterine multinodular fibromatosis. A transvaginal ultrasound showed the presence of an intrauterine mass, for which she underwent a total laparoscopic hysterectomy, bilateral adnexectomy, pelvic and paraaortic lymph node biopsies, and omentectomy. The pathology report described a 7 cm diameter, stage IB uterine mass consistent with grade 3 uLMS. Histology showed smooth muscle actin (SMA) positive and desmin positive spindle cells with marked cytological atypia and 15 mitoses per 10 high power fields (HPF). Tumor necrosis was below 50% and no tumoral invasion was found in the uterine serous and omentum. Postoperatively, the patient received adjuvant chemotherapy with epirubicine plus ifosfamide.

**Figure 1:**
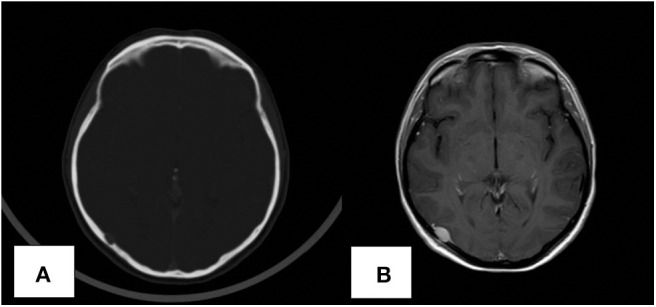
Computed tomography (CT) scan revealing the presence of the skull lesion **(A)**. MRI image of the solitary skull metastatic lesion **(B)**.

Clinical and imaging follow up showed no evidence of recurrence until June 2019, when a total body computed tomography (CT) scan revealed an osteolytic lesion in the right temporal-occipital region, 8 mm in diameter and positive to bone scintigraphy. Physical and neurological examination were normal. The subsequent cranial magnetic resonance imaging (MRI) confirmed the presence of the osteolytic temporal-occipital lesion, with epidural extension and partial compression of the underlying brain parenchyma (Figures 1A,B). MRI showed homogeneous signal on T2-weighted imaging and hypo-intensity on T1 weighted images; CT-PET was negative for other metastatic lesions.

**Figure 2:**
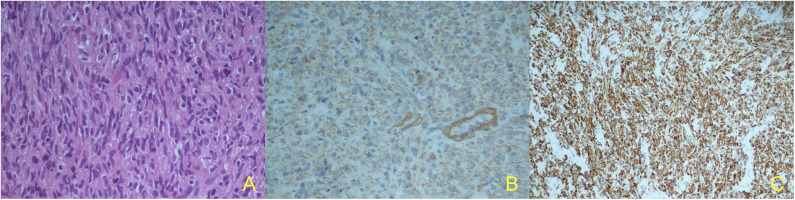
Histological specimen: marked cellular pleomorphism, nuclear atypia, mitotic figures. Hematoxylin and eosin (H&E stain), X40 **(A)**; Immunohistochemical findings: strong cell positivity for alfa-smooth muscle actin (a-SMA), X40 **(B)** and strong cell positivity for vimentin, X20 **(C)**.

In October 2019, she underwent a right craniotomy with removal of the lesion and the involved scalp; epidural extension of the tumor was confirmed. Although surgery resulted in removal of all macroscopic disease, microscopic margins were positive (R1). Histological exam showed the presence of spindle cells with moderate pleomorphism, 15 mitotic figures per 10 HPF (Figure 2A), and strong positivity for smooth muscle actin (SMA) (Figure 2B), vimentin, and desmin in immunohistochemistry (Figure 2C). All these findings were consistent with metastatic uLMS. Given the extreme rarity of skull recurrence of uLMS, a multidisciplinary approach was followed. In absence of other metastatic foci and given the R1 resection and the overall limited benefit of chemotherapy as systemic treatment in a patient who had already undergone anthracycline treatment reaching a high cumulative dose, radiation treatment was performed (3,500 centigray—cGy—in 5 fractions of 700 cGy). The patient is currently alive, without evidence of extracranial disease.

## Discussion

Although hysterectomy offers the best chance of prolonged survival and it is considered as the standard treatment for uLMS, post-operative recurrence rate ranges from 50 to 70%, with a very poor prognosis (11). Lungs represent the most common site of recurrence, followed by peritoneum, liver, vertebral and long bones. However, a heightened awareness of atypical patterns of recurrence and the recent progress in imaging techniques have resulted in a marked increase in the detection of unusual and rare sites of uLMS relapse (12–15). These findings are not exclusively limited to case reports, with retrospective analysis suggesting the presence of less common sites of metastases whose knowledge could reduce diagnostic delay and improve survival (14). With regards to atypical sites of recurrence, a wide range of time intervals between surgical resection of the primary tumor and metastasis onset has been reported, highlighting the need for long-term follow-up and adequate diagnostic confirmation (15).

To the best of our knowledge, two cases of skull recurrence of uLMS have been reported in literature to date (16, 17) (Table 1). In the first case, a 54-year-old woman presented with a cranial mass 5 years after radical hysterectomy for uLMS. No additional sites of metastasis were reported. Although radical surgery was performed, the patient developed disease progression after 1 year, with multiple bone and lung metastases; she died of pulmonary dysfunction after 12 months. In the other one, a 63-year-old woman presented a bulging mass over her left parietal region 14 months after radical surgery for uLMS. Despite the patient undergoing total tumor resection, she expired after 4 months.

**Table 1:** Summary of three reported cases of skull metastases from uterine leiomyosarcoma.

**Authors/ year**	**Age at uLMS diagnosis**	**Adjuvant treatment after surgery of primary tumor**	**Interval from diagnosis of primary lesion to brain metastasis (months)**	**Site**	**Surgery**	**Adjuvant treatment after cranial surgery**	**1st line CHT**	**Interval from brain metastasis to death (months)**
Uchino et al. (16)	49	Not performed	60	Frontal bone	R0 resection	Adriamycin, Vincristine, Cisplatin	No	24
Yip et al. (17)	62	CHT (schedule not specified)	15	Left parietal bone	R0 resection	Not performed	No	4 (not uLMS-related death)
Present report	38	Epirubicine—Ifosfamide	18	Right temporal—occipital bone	R1 resection	Radiation therapy (3,500 cGy in five fractions of 700 cGy)	No	NA

*uLMS, uterine leiomyosarcoma; CHT, chemotherapy; R0, radical surgery, with no cancer cells seen microscopically at the resection margin; R1, cancer cells present microscopically at the resection margin (microscopic positive margin); NA, not available; cGy, centiGray*.

Signs and symptoms of skull metastases appears as highly non-specific, depending on tumor extension. This may lead to differential diagnosis, especially after several years of disease-free survival (18). In this context, clinical suspicion and complete physical examination are necessary but not sufficient tools. Another key element is the imaging technique chosen during follow-up of completely resected uLMS. Current guidelines recommend chest, abdomen, and pelvis CT scan during postoperative follow-up, however, a cranial CT scan is rarely included (19).

If metastatic uLMS is certainly associated with poor prognosis, from a theoretical point of view solitary metastasis may show better clinical outcomes. All the patients reported in literature to date, including our case, showed solitary skull recurrence in the absence of other metastatic foci of uMLS. Given the rarity of metastasis to this region, there is a lack of evidence supporting a specific treatment of intracranial metastases from uLMS, whether metastatic site is brain or skull (20). Management should therefore be personalized depending on clinical condition, site, and size of metastasis (9). Maximum treatment should be given to patients with good performance status and who are suitable for surgery, chemotherapy, and/or radiotherapy. The heterogeneity of recurrence patterns, the lack of an established treatment in this setting, and the prognostic burden of metastatic uLMS justify the need for a multidisciplinary team (MDT) in order to provide quicker time to treatment after diagnosis and to translate MDT approach into better survival outcomes. However, given the rarity of intracranial recurrence, it is worth it to underline that the optimal MDT modalities, sequence, and timing of treatments are all yet to be defined.

## Conclusions

Diagnosis of skull recurrence of uLMS requires a high index of suspicion. Its early recognition can be extremely difficult, especially in asymptomatic, healthy patients in which cranial CT scan is often not performed during post-surgery follow-up. There is lack of evidence for choosing the best therapeutic strategy in these patients and appropriate treatment of intracranial recurrences is yet to be defined.

The presented report is the third documented case of skull metastasis from uLMS suggesting the importance of improving awareness of this extremely rare occurrence.

Therefore, we encourage the collection of larger series in order to provide further information about cranial recurrences of uLMS, taking into account the paucity of data currently available in literature and the frequently unpredictable behavior of this rare and highly lethal disease.

## Data Availability Statement

All datasets generated for this study are included in the article/supplementary material.

## Ethics Statement

Written informed consent was obtained from the individual(s) for the publication of any potentially identifiable images or data included in this article.

## Author Contributions

AR and MCN have made substantial contributions to conception of the study, interpreted the patient data, and drafted the manuscript. VR performed surgery and has been involved in drafting the manuscript. CG performed the histological and pathologic examination of the surgical tissue and has been involved in drafting figures. AP and PD have been involved in drafting and revising the manuscript. GF, DB, and MD performed radiotherapy and have been involved in drafting and revising the manuscript. JM performed imaging and has been involved in drafting the manuscript. MP has made substantial contributions to conception of the study, interpreted the patient data, and drafted the manuscript. MN was the major contributor in concept and designed and revised the manuscript. All authors read and approved the final version of the manuscript.

## Conflict of Interest

The authors declare that the research was conducted in the absence of any commercial or financial relationships that could be construed as a potential conflict of interest.

## Abbreviations

cGy: centigray
CT: computed tomography
HPF: high power fields
MDT: multidisciplinary team
MRI: magnetic resonance imaging
OS: overall survival
uLMS: uterine leiomyosarcoma
US: uterine sarcoma.
